# Supplementary material for: Factors that contributed to Ontario adults’ mental health during the first 16 months of the COVID-19 pandemic: a decision tree analysis
Source: PeerJ. 2024 Mar 29;12:e17193. doi: 10.7717/peerj.17193 (PMC10984169; doi:10.7717/peerj.17193)
Supplement: Supplemental Information 1 — Note. aOpen-ended question; bNo participants selected “Not listed please specify” or “I prefer not to answer”, as such, these categories were not included in the analysis; cNot all ethnic categories are presented, only those participants self-selected into. [file peerj-12-17193-s001.docx]

**Supplemental File 1. Summary of Items, Response Options, and Variable Type Used in Analysis**

| **Items** | **Response Options** | **Use in Analysis** |
| --- | --- | --- |
| **Dependent Variable** | | |
| Mental Health | 0-100 | Binary: mental health problems (0-76); no mental health problems (77-100) |
| **Demographic Independent Variables** | | |
| Age | N/A^a^ | Continuous |
| Gender | Male, female, non-binary, not listed please specify, I prefer not to answer | Nominal: male; female; non-binary^a^ |
| Marital Status | Single; married/common law/engaged; divorced/separated; widowed | Nominal: single; co-habiting |
| Ethnicity | Arab; Black; Caucasian (White); Chinese; Filipino; Indigenous; Japanese; Korean; Latin American; Metis; Multiracial; South Asian; Southeast Asian^c^ | Nominal: white; person of colour |
| Employment | Employed full-time; employed part-time; unemployed; casual; other | Nominal: employed; casual/part-time; unemployed; other (e.g., lay-off due to COVID-19) |
| Education | Less than high school; high school completed; community college and/or journeyman apprenticeship completed; university undergraduate degree completed; university graduate degree or higher completed; other | Nominal: high school; community college/journeyman; university; other |
| **Health Behaviour/Outcome Independent Variables** | | |
| Sleep | 0-21 | Binary: good sleep (0-4); poor sleep (5-21) |
| Wellbeing | 0-100 | Binary: poor wellbeing (0-69); good wellbeing (70-100) |
| Recreational-Related Physical Activity | N/A^a^ | Binary: meeting (150+ mins/wk; not meeting (0-149 mins/wk) |
| Sedentary Time | N/A^a^ | Binary: meeting (0-8 hrs/day), not meeting (9+ hrs/day) |
| Screen Time | N/A^a^ | Continuous |

*Note*. ^a^Open-ended question; ^b^No participants selected “Not listed please specify” or “I prefer not to answer”, as such, these categories were not included in the analysis; ^c^Not all ethnic categories are presented, only those participants self-selected into.
