# Supplementary material for: Factors that contributed to Ontario adults’ mental health during the first 16 months of the COVID-19 pandemic: a decision tree analysis
Source: PeerJ. 2024 Mar 29;12:e17193. doi: 10.7717/peerj.17193 (PMC10984169; doi:10.7717/peerj.17193)
Supplement: Supplemental Information 2 — Checklist of items that should be included in reports of observational studies [file peerj-12-17193-s002.docx]

STROBE Statement—checklist of items that should be included in reports of observational studies

|  | Item No. | Recommendation | Page  No. | Relevant text from manuscript |
| --- | --- | --- | --- | --- |
| **Title and abstract** | 1 | (*a*) Indicate the study’s design with a commonly used term in the title or the abstract | 1 | Factors That Contributed to Ontario Adults’ Mental Health During the First 16 Months of the COVID-19 Pandemic: A Decision Tree Analysis |
|  |  | (*b*) Provide in the abstract an informative and balanced summary of what was done and what was found | 3 | Data were collected via online surveys at two time points: April-July 2020 and July-August 2021; 2,188 adults (*M*_age_ = 43.15 years; *SD* = 8.82) participated. Surveys included a demographic questionnaire and four previously validated tools to measure participants’ mental health, subjective wellbeing, physical activity and sedentary behaviour, and sleep. A decision tree was generated at each time point for those with *mental health problems*, and those with *no mental health problems*. Results showed that subjective wellbeing was the biggest contributor to mental health status. Characteristics associated with *no mental health problems* among adults included having good wellbeing, being a good sleeper (quantity, quality, and patterns of sleep), and being over the age of 42. Characteristics associated with *mental health problems* included having poor wellbeing and being a poor sleeper. Findings revealed that specific characteristics interacted to contribute to adults’ mental health status during the first 16 months of the COVID-19 pandemic. |
| Introduction | | | |  |
| Background/rationale | 2 | Explain the scientific background and rationale for the investigation being reported | 8-9 | It is clear, based on the above review of literature, that the COVID-19 pandemic has negatively impacted the mental health of adults globally; however, less is known regarding the characteristics that contributed to some people having mental health problems during the first 16 months of the COVID-19 pandemic in Ontario, Canada, while others did not. Understanding this is critical, as the first 16 months of the pandemic included ongoing adaptations and restrictions in attempting to mitigate the severity of health issues (Canadian Institute for Health Information, 2022). Of necessity, most of the foci during this period were on developing vaccinations and advocating for preventive measures like physical distancing (Canadian Institute for Health Information, 2022; Chu et al., 2020; Government of Canada, 2020). Of the provinces in Canada, Ontario was among few with strict preventative measures in place during the early months of the pandemic (Dekker & Macdonald, 2022). Thus, understanding the characteristics that contributed to some adults’ having mental health problems during this timeframe, while others did not, is instructive to inform the development of programs/interventions aimed at mitigating the negative impact of the COVID-19 pandemic on adults’ mental health, as well as creating tailored interventions, responsive to the characteristics of those who had the highest incidence of mental health problems. |
| Objectives | 3 | State specific objectives, including any prespecified hypotheses | 9 | As such, the purpose of this study was to generate profiles of Ontario adults who were more or less at risk for the development of mental health problems during the first 16-months of the COVID-19 pandemic. |
| Methods | | | |  |
| Study design | 4 | Present key elements of study design early in the paper | 9 | This study represents part of an ongoing, longitudinal, survey-based research project titled Health Outcomes for adults during and following the COVID-19 PandEmic (HOPE), which was designed to assess adults’ lifestyle-related health behaviours and outcomes, including physical activity, sedentary behaviour, sleep, diet, mental health, wellbeing, and prosocial behaviour, during and following the COVID-19 pandemic in Ontario, Canada (Shillington et al., 2021; Shillington et al., 2022a; Shillington et al., 2022b; Shillington et al., 2023). |
| Setting | 5 | Describe the setting, locations, and relevant dates, including periods of recruitment, exposure, follow-up, and data collection | 9 | The current paper includes data collected at time point 1 (April 24–July 13, 2020) and time point 3 (July 29–August 30, 2021) to generate profiles of Ontario adults who were at more or less risk for the development of mental health problems during the first 16-months of the COVID-19 pandemic. |
| Participants | 6 | (*a*) *Cohort study*—Give the eligibility criteria, and the sources and methods of selection of participants. Describe methods of follow-up  *Case-control study*—Give the eligibility criteria, and the sources and methods of case ascertainment and control selection. Give the rationale for the choice of cases and controls  *Cross-sectional study*—Give the eligibility criteria, and the sources and methods of selection of participants | 9-10 | Study participants had to be: (1) an Ontario resident; (2) between the ages of 30–59 years at baseline, as individuals within this age range are at highest risk for losing years of healthy life due to chronic disease (WHO, 2005); and (3) able to read and write in English. Interested individuals were asked to click the link in the study advertisement, which directed them to an online survey administered via Qualtrics, that included the letter of information, eligibility questions, consent, and the time 1 questionnaires. To provide electronic consent, participants were asked to click “I consent to begin the study”, acknowledging that they understood the terms and conditions of participating in the study and were making an informed decision to participate. |
|  |  | (*b*) *Cohort study*—For matched studies, give matching criteria and number of exposed and unexposed  *Case-control study*—For matched studies, give matching criteria and the number of controls per case | N/A | N/A |
| Variables | 7 | Clearly define all outcomes, exposures, predictors, potential confounders, and effect modifiers. Give diagnostic criteria, if applicable | 10-13 | Exposures. There were 11 explanatory variables explored in the analysis. These included demographic variables (n = 6; gender, age, ethnicity, employment, marital status, and education) which were collected at time 1, and health behaviours/outcomes (n = 5; sleep, wellbeing, physical activity, sedentary time, and screen time) which were collected at times 1 and 3. Health behaviours/outcomes were assessed using previously validated scales including the Pittsburgh Sleep Quality Index (PSQI; Buysse et al., 1988), the Personal Wellbeing Index-Adult (PWI-A; International Wellbeing Group, 2013), and the Global Physical Activity Questionnaire (GPAQ; Bull et al., 2009), described below.  The PSQI has been previously validated (Cronbach α = 0.83) and is used to measure adults’ quantity, quality, and patterns of sleep on seven domains: (1) subjective sleep quality; (2) sleep latency; (3) sleep duration; (4) habitual sleep efficiency; (5) sleep disturbances; (6) use of sleep medication; and (7) daytime dysfunction (Buysse et al., 1988). The seven domains are then summed to yield a total score ranging from 0-21, wherein a score greater than 5 classifies participants as “poor sleepers” (Buysse et al., 1988). The total score was used in the current study, where participants were coded as 0 if they scored between 0 and 4 (indicating “good sleepers”) or 1 if they scored between 5 and 21 (indicating “poor sleepers”).  The PWI-A has been previously validated (Cronbach’s α = 0.70-0.85) and is used to measure subjective wellbeing (International Wellbeing Group, 2013). The scale includes 7 items that corresponded to quality of life domains including: (1) standard of living; (2) health; (3) achievement in life; (4) relationships; (5) safety; (6) community-connectedness; and (7) future security (International Wellbeing Group, 2013). It also includes two additional (optional) items: (1) satisfaction with life as a whole; and (2) spirituality/religion (International Wellbeing Group, 2013). Data can be interpreted at the individual (domain) level; alternatively, the domains can be summed to yield a total wellbeing score (excluding satisfaction with life as a whole, per the scoring protocol; International Wellbeing Group, 2013). For the purpose of this study, the domains were summed, wherein a score less than 70 indicated poor wellbeing (International Wellbeing Group, 2013). As such, participants were coded as 0 if they scored between 0 and 69 (indicating poor wellbeing) or 1 if they scored between 70-100 (indicating good wellbeing).  The GPAQ has been previously validated (Bull et al., 2009) and is used to measure physical activity at the population level. This scale includes four domains: (1) activity at work; (2) travel to and from places; (3) recreational activities; and (4) sedentary behaviour (Bull et al., 2009). For the purpose of this study, the domains of recreational activities and sedentary behaviour were used. To yield a total score for recreational-related physical activity (moderate to vigorous physical activity; MVPA), data from the following questions were used: (1) “In a typical week, on how many days do you do vigorous-intensity sports, fitness or recreational (leisure) activities?”; (2) “How much time do you spend doing vigorous-intensity sports, fitness or recreational activities on a typical day?”; (3) “In a typical week, on how many days do you do moderate-intensity sports, fitness or recreational (leisure) activities?”; and (4) “How much time do you spend doing moderate-intensity sports, fitness or recreational activities on a typical day?” The total score for recreational-related physical activity was in minutes per week, wherein participants who engaged in 0-149 minutes of recreational-related physical activity per week were coded as 0 (not meeting recommended amount of MVPA) and participants who engaged in 150 minutes or greater of recreational-related physical activity per week were coded as 1 (meeting recommended MVPA), per Canada’s 24-Hour Movement Guidelines for adults (Canadian Society for Exercise Physiology, 2020). With respect to sedentary behaviour, participants were asked “How much time do you usually spend sitting or reclining on a typical day?” Participants who engaged in more than 8 hours of sedentary pursuits per day were coded as 0 (not meeting recommended sedentary behaviour guidelines) and participants who engaged in 8 hours or less of sedentary pursuits per day were coded as 1 (meeting recommended sedentary behaviour guidelines), again as outlined in Canada’s 24-Hour Movement Guidelines (Canadian Society for Exercise Physiology, 2020). Lastly, to measure participants’ screen use, the following question was used: “How much time do you usually spend watching TV or using a computer, tablet or smartphone on a typical day?” This item was not manually coded, as it was a continuous variable and thus a standard median split was used (calculated via SPSS version 29.0). Supplementary 1 outlines the response scale for each variable as well as variable type (e.g., nominal and ordinal) and number of levels.  Outcome. The outcome variable in the current study was mental health, which was assessed using the Mental Health Inventory-5 (MHI-5; Berwick et al., 1991). The MHI-5 has been previously validated and measures mental health status using 5 items: general positive affect (2 items); anxiety (1 item); depression (1 item); and behavioural/emotional control (1 item; Berwick et al., 1991). The items are then summed to yield a total mental health score. While the authors of the scale did not establish a cut score, researchers have used a cut score of 76 and below to indicate mental health problems (Kelly et al., 2008). As such, participants were coded as 0 if they scored between 0 and 76 (indicating mental health problems) or 1 if they scored between 77-100 (indicating no mental health problems). |
| Data sources/ measurement | 8* | For each variable of interest, give sources of data and details of methods of assessment (measurement). Describe comparability of assessment methods if there is more than one group | 10-13 | See text above. |
| Bias | 9 | Describe any efforts to address potential sources of bias | N/A | N/A |
| Study size | 10 | Explain how the study size was arrived at | N/A | N/A |

Continued on next page

| Quantitative variables | 11 | Explain how quantitative variables were handled in the analyses. If applicable, describe which groupings were chosen and why | 10-14 | See text above (‘Variables’) and below (‘Statistical Methods’) for reference. |
| --- | --- | --- | --- | --- |
| Statistical methods | 12 | (*a*) Describe all statistical methods, including those used to control for confounding | 13-14 | Decision tree modeling has been applied in the fields of medicine and public health (Batterham et al., 2009; Camp & Slattery, 2002; Jung et al., 2015) and can be used to understand features and extract patterns in large datasets (Myles et al., 2004). It is a commonly used method for “establishing classification systems based on multiple covariates or for developing prediction algorithms for a target variable” (Song & Lu, 2015, p.130). Profiles in the current study (mental health problems vs. no mental health problems) were generated based on two broad categories of variables: (1) demographics (gender, age, ethnicity, employment, marital status, and education); and (2) health behaviours/outcomes (sleep, wellbeing, physical activity, sedentary time, and screen time). Some of the health behaviour/outcomes were dichotomized using cut-scores (noted above) and to this end, we relied on a set of binary rules to calculate a target value related to our study objective. No a priori hypotheses were put forward as decision tree modeling is a data-driven analysis and requires no formal theoretical structure (Guerrero et al., 2020).    Decision tree models for the current study were generated using the exhaustive chi-square automatic interaction detector (CHAID) algorithm (McArdle & Ritschard, 2014). Exhaustive CHAID is commonly used when examining large datasets and involves splitting the data into “mutually exclusive, exhaustive, subsets that best describe the dependent variable” (Kass, 1980, p. 199). This method has a number of strengths including: (1) it is non-parametric; (2) it is robust against issues regarding missing data and outliers; and (3) all types of variables (continuous, ordinal, categorical) can be included (Merkle & Shaffer, 2011). The exhaustive CHAID method begins with a root (or “parent”) node that splits into two or more mutually exclusive subsets (“child nodes”; Song & Lu, 2015). Nodes continue to split until pre-determined homogeneity or stopping criteria are met (Song & Lu, 2015). The following statistical model specifications and stopping criteria were applied in the current study: (1) the significance level for splitting nodes was set at p < 0.05; (2) the Bonferroni method was used to obtain the significant values of adjustment; (3) the minimum change in expected cell frequencies was 0.001; (4) Pearson’s X2 was used; (5) model depth was set at 3; (6) the minimum number of cases in parent nodes was set at 100 and in child nodes was set at 50; (7) cross-validation (10-folds) was used to assess the tree structure; and, (8) the mis-classification risk was calculated as a measure of model reliability. Missing values were handled using multiple imputation and data were analyzed in SPSS. Two models were generated, one utilizing time point 1 data and a second utilizing time point 3 data. All participants were included in the time point 1 decision tree model (N = 2,188); one participant was excluded from the time point 3 decision tree model due to invalid/spurious data. |
|  |  | (*b*) Describe any methods used to examine subgroups and interactions | N/A | N/A |
|  |  | (*c*) Explain how missing data were addressed | 13-14 | See text above. |
|  |  | (*d*) *Cohort study*—If applicable, explain how loss to follow-up was addressed  *Case-control study*—If applicable, explain how matching of cases and controls was addressed  *Cross-sectional study*—If applicable, describe analytical methods taking account of sampling strategy | 13-14 | See text above. |
|  |  | (*e*) Describe any sensitivity analyses | N/A | N/A |
| Results | | | | |
| Participants | 13* | (a) Report numbers of individuals at each stage of study—eg numbers potentially eligible, examined for eligibility, confirmed eligible, included in the study, completing follow-up, and analysed | 14 | A total of 2,188 individuals participated in the study. |
|  |  | (b) Give reasons for non-participation at each stage | N/A | N/A |
|  |  | (c) Consider use of a flow diagram | N/A | N/A |
| Descriptive data | 14* | (a) Give characteristics of study participants (eg demographic, clinical, social) and information on exposures and potential confounders | 14-15 | The mean age of participants was 43.15 years (SD = 8.82), with the majority identifying as female (n = 1,743; 89.55%), of European ancestry (n = 1,789; 91.55%), and having a university degree or higher (n = 1,123; 57.21%). Additionally, most participants in the study were married, common law, or engaged (n = 1,535; 78.20%). Full demographic details have been published elsewhere (Shillington et al., 2023). |
|  |  | (b) Indicate number of participants with missing data for each variable of interest | 14 | Missing values were handled using multiple imputation and data were analyzed in SPSS. Two models were generated, one utilizing time point 1 data and a second utilizing time point 3 data. All participants were included in the time point 1 decision tree model (N = 2,188); one participant was excluded from the time point 3 decision tree model due to invalid/spurious data. |
|  |  | (c) *Cohort study*—Summarise follow-up time (eg, average and total amount) | N/A | N/A |
| Outcome data | 15* | *Cohort study*—Report numbers of outcome events or summary measures over time | 15, 18 | ***Time Point 1 (April 24–July 13, 2020) Decision Tree***  Figure 1 shows the final 3-level model at time 1, comprising 13 nodes, 7 of which were terminal subgroups (i.e., nodes that do not split any further). Four predictor variables reached significance (wellbeing, sleep, physical activity, age) and were selected because they best differentiated adults who had mental health problems (79.6%) from those who did not (20.4%) based on the cut scores outlined above. The first level of the tree was split into 2 initial branches according to participants’ perceived wellbeing, meaning that this variable was the best predictor of mental health status (*mental health problems* or *no mental health problems*). The *no mental health problems* group included participants who had good wellbeing (Node 2), were classified as good sleepers (Node 6), and who were over the age of 42 (Node 12; 72.0% *no mental health problems*). The probability decreased when participants were 42 years of age or younger (Node 11; 45.3% *no mental health problems*). Those in the *mental health problems* group had poor wellbeing (Node 1) and were classified as poor sleepers (Node 3; 93.2% *mental health problems*). The probability decreased when participants were classified as good sleepers (Node 4) and were not meeting the MVPA recommendations (Node 8; 87.0% *mental health problems*), and when they were classified as good sleepers (Node 4) and were meeting the MVPA recommendations (Node 7; 75.6% *mental health problems*). Decision rules for the prediction of mental health problems at time point 1 are presented in Table 1, which also shows detailed “IF–THEN” rules.  ***Time Point 3 (July 29–August 30, 2021) Decision Tree***  Figure 2 shows the final 3-level model at time 3, comprising 8 nodes, 5 of which were terminal subgroups. Three predictor variables reached significance (wellbeing, sleep, age) and were selected because they best differentiated adults who self-reported mental health problems (76.1%) from those who did not (23.9%). The first level of the tree was split into 2 initial branches according to participants’ perceived wellbeing, meaning that this variable was the best predictor of mental health status (mental health problems or no mental health problems). The no mental health problems group included participants who had good wellbeing (Node 2) and were classified as good sleepers (Node 6; 63.4% no mental health problems). The probability decreased when participants were classified as poor sleepers (Node 5) and were over the age of 52 (Node 8; 60.2% no mental health problems) and when they were classified as poor sleepers (Node 5) and were 52 years of age or younger (Node 7; 39.9% no mental health problems). Participants in the mental health problems group included participants who had poor wellbeing (Node 1) and were classified as poor sleepers (Node 3; 88.7% mental health problems). The probability decreased when participants were classified as good sleepers (Node 4; 75.8% mental health problems). Decision rules for the prediction of mental health problems at time point 3 are presented in Table 2. |
|  |  | *Case-control study—*Report numbers in each exposure category, or summary measures of exposure | N/A | N/A |
|  |  | *Cross-sectional study—*Report numbers of outcome events or summary measures | N/A | N/A |
| Main results | 16 | (*a*) Give unadjusted estimates and, if applicable, confounder-adjusted estimates and their precision (eg, 95% confidence interval). Make clear which confounders were adjusted for and why they were included | N/A | N/A |
|  |  | (*b*) Report category boundaries when continuous variables were categorized | N/A | N/A |
|  |  | (*c*) If relevant, consider translating estimates of relative risk into absolute risk for a meaningful time period | N/A | N/A |

Continued on next page

| Other analyses | 17 | Report other analyses done—eg analyses of subgroups and interactions, and sensitivity analyses | N/A | N/A |
| --- | --- | --- | --- | --- |
| Discussion | | | | |
| Key results | 18 | Summarise key results with reference to study objectives | 21 | The purpose of this paper was to use decision tree modeling to generate profiles of adults who were more or less at risk for the development of mental health problems during the first 16-months of the COVID-19 pandemic in Ontario, Canada. The models yielded profiles based on demographic characteristics and health behaviours/outcomes. From April 24–July 13, 2020 (time point 1), characteristics of those in the ‘no mental health problems’ group included having good wellbeing, being a good sleeper, and being over the age of 42, while characteristics of those in the ‘mental health problems’ group included having poor wellbeing and being a poor sleeper. From July 29–August 30, 2021(time point 3), characteristics associated with no mental health problems included having good wellbeing and being a good sleeper, while characteristics associated with mental health problems included having poor wellbeing and being a poor sleeper. |
| Limitations | 19 | Discuss limitations of the study, taking into account sources of potential bias or imprecision. Discuss both direction and magnitude of any potential bias | 25 | This study is not without limitations. First, while the sample size was large (N = >2,000) there was attrition between time points. To handle missing data due to attrition and maintain sample size, multiple imputation was used. Second, the study lacks generalizability because the sample was primarily comprised of White, female-identifying individuals of high socioeconomic status. This likely influenced study findings, as no demographic characteristics (aside from age, which was a continuous variable) were included in the decision tree models. It is possible that this was due to the disproportionate sample sizes for each group/category. As such, while gender, ethnicity, employment, marital status, and education were not strong predictors of mental health status in the current sample, this is not to say that such variables do not contribute to an individual’s mental health status in general. Given that the COVID-19 pandemic has negatively affected minoritized individuals and those of low socioeconomic status, findings from the current study are not representative of such voices and experiences. In the future, researchers might consider stratifying their sample by targeting groups of diverse ethnic origins, genders, and socioeconomic status in order to achieve greater diversity and representation among the population. Lastly, it is worth noting that wherever possible, authors relied on pre-established cut-scores (e.g., PSQI, Buysse et al., 1988; PWI-A, International Wellbeing Group, 2013) to dichotomize the health behaviour/outcome variables for the decision tree models. However, in instances where such thresholds were not available, such was the case with the MHI-5 (Berwick et al., 1991), our approach to dichotomizing the outcome variables was informed by recommendations in the literature (Kelly et al., 2008). |
| Interpretation | 20 | Give a cautious overall interpretation of results considering objectives, limitations, multiplicity of analyses, results from similar studies, and other relevant evidence | 21-24 | Paragraphs 2-4 |
| Generalisability | 21 | Discuss the generalisability (external validity) of the study results | 25 | See text above. |
| Other information | |  | | |
| Funding | 22 | Give the source of funding and the role of the funders for the present study and, if applicable, for the original study on which the present article is based | 2 | No funding was received. |

*Give information separately for cases and controls in case-control studies and, if applicable, for exposed and unexposed groups in cohort and cross-sectional studies.

**Note:** An Explanation and Elaboration article discusses each checklist item and gives methodological background and published examples of transparent reporting. The STROBE checklist is best used in conjunction with this article (freely available on the Web sites of PLoS Medicine at http://www.plosmedicine.org/, Annals of Internal Medicine at http://www.annals.org/, and Epidemiology at http://www.epidem.com/). Information on the STROBE Initiative is available at www.strobe-statement.org.
