# Supplementary material for: Factors that contributed to Ontario adults’ mental health during the first 16 months of the COVID-19 pandemic: a decision tree analysis
Source: PeerJ. 2024 Mar 29;12:e17193. doi: 10.7717/peerj.17193 (PMC10984169; doi:10.7717/peerj.17193)
Supplement: Supplemental Information 3 [file peerj-12-17193-s003.docx]

**Supplemental File 3**

**Test Instrument Permissions**

The Personal Wellbeing Index-Adult (PWI-A; International Wellbeing Group, 2013) and the Mental Health Inventory-5 (MHI-5; Berwick et al., 1991) were used accordance with a published license and were available in the public domain.

The PWI-A was made available through the following public domains: <https://www.acqol.com.au/uploads/pwi-a/pwi-a-english.pdf> and <https://www.acqol.com.au/instruments>.

The MHI-5 was made available through the following public domains: <https://www.jstor.org/stable/3766262?seq=8>, <https://link.springer.com/article/10.1186/1756-0500-4-100>
